# Nature-Inspired Photocatalytic Hydrogen Production with a Flavin Photosensitizer

**DOI:** 10.1021/acsomega.3c07458

**Published:** 2024-01-26

**Authors:** Lucia Ivanová, Jan Truksa, Dong Ryeol Whang, Niyazi Serdar Sariciftci, Cigdem Yumusak, Jozef Krajčovič

**Affiliations:** † Faculty of Chemistry, Brno University of Technology, Purkyňova 118, CZ-612 00 Brno, Czech Republic; ‡ Department of Advanced Materials, Hannam University, 70 Hannamro, Daedeok-Gu, Daejeon 34430, Republic of Korea; § Linz Institute for Organic Solar Cells (LIOS), Institute of Physical Chemistry, Johannes Kepler University Linz, Altenberger Straße 69, 4040 Linz, Austria

## Abstract

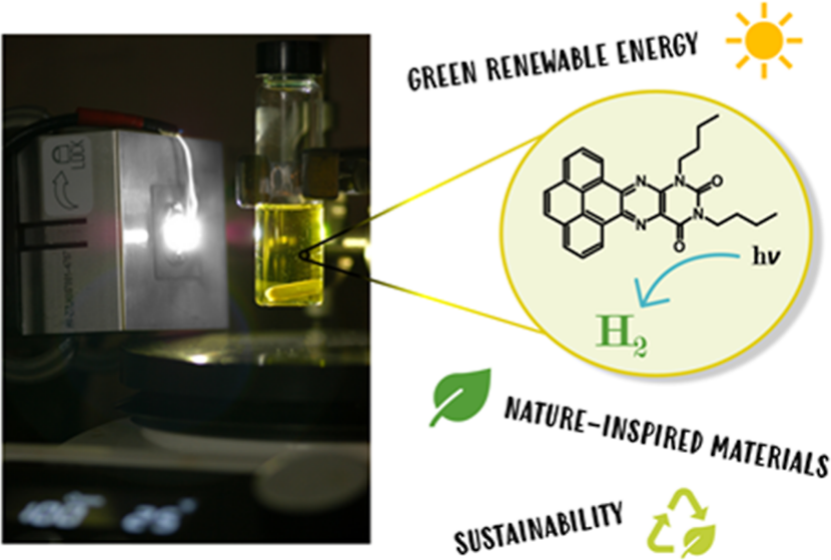

Green hydrogen, by definition, must be produced with renewable energy sources without using fossil fuels. To transform the energy system, we need a fully sustainable production of green and renewable energy as well as the introduction of such “solar fuels” to tackle the chemical storage aspect of renewable energies. Conventional electrolysis of water splitting into oxygen and hydrogen gases is a clean and nonfossil method, but the use of massive noble-metal electrodes makes it expensive. Direct photocatalytic hydrogen evolution in water is an ideal approach, but an industrial scale is not available yet. In this paper, we intend to introduce flavins as metal-free organic photosensitizers for photoinduced reduction processes. Specifically, a flavin photosensitizer was employed for the photocatalytic evolution of hydrogen gas in aqueous media. The ratio of photosensitizer to cocatalyst concentration has been found to affect the efficiency of the hydrogen evolution reaction. Since flavins are nature-inspired molecules (like vitamin B2) with easily tunable properties through structure modification, this family of compounds opens the door for new possibilities in sustainable green hydrogen production.

## Introduction

Considering the prevalent use of fossil fuels, the increase in the amount of combustion emissions in the atmosphere, and the weather-dependent reliability of renewable sources (e.g., wind power plants), there is a need for emissionless fuel with a high energy density; the search for such nonfossil-based power sources is a top priority within the scientific community. In this context, hydrogen, whose production is powered by renewable energy without direct CO_2_ emission (often referred to as green hydrogen), could become one of the alternative fuels of the future due to a high gravimetric energy density of 122 kJ g^–1^.^1,2^ This could be a viable alternative to traditional energy storage methods for electrochemical batteries. In order to reach carbon neutrality in energy systems, the global production capacity for green hydrogen must increase by several orders of magnitude in the next decade.^3^ This is a massive challenge, scientifically, technically, as well as politically.

Hydrogen today is produced by the reverse Sabatier reaction (using direct steam reforming and/or water gas shift reaction, etc.) by stripping the hydrogens from fossil methane gas. This is not sustainable and produces CO_2_ in the process.

Conventional electrolysis of water splitting into oxygen and hydrogen gases is a clean and nonfossil method, but the use of massive noble metal electrodes makes it expensive.

Currently, the main process on the industrial scale is electrolytic hydrogen production by water splitting. Today, the electrical power for H_2_ production by water electrolysis comes mainly from nonrenewable fossil fuels, even in developed countries. If we address the question of the source of electrical power, electrification today is mostly based on fossil fuels, and then electrolysis is also fossil fuel-related and -dependent.

Furthermore, the main cocatalysts used therein, such as platinum, are often costly or toxic.^4,5^ For these and many other reasons, looking for other effective alternative components for an environmentally friendly and fully renewable technology is necessary.

Direct photocatalysis has the advantage of directly utilizing solar energy, making it more ecologically valuable in remote places and small-scale applications. Today’s drawback is that the overall conversion efficiency from “light energy converted into stored chemical energy” of direct photocatalysis is still lower than the indirect conversion using high-efficiency photovoltaics and subsequent electrocatalysis. That is scientifically a grand challenge to make direct photocatalysis more efficient. Many papers, books, and reports have been published in the last 50 years on the topic of “artificial photosynthesis”.^2,6−9^

Prevalently, molecular-based H_2_-evolving systems are multicomponent. Arguably, the development of the photosensitizer is a considerable research challenge as it plays the important role of capturing initial solar irradiation and consequent photoexcited electron transfer through the system to a photochemically unreactive substrate. In the view of light harvesters, inorganic semiconductors (transition-metal oxides or hydroxides and their derivatives);^10,11^ organic polymeric semiconductors;^12^ graphitic materials;^13^ metal–organic frameworks with photoresponsive metal ions surrounded with organic ligands;^14^ organic dyes;^15^ and organometallic complexes are several established divisions.^16,17^ The value of the photosensitizer increases with the abundance of the material, good photostability and stability in neutral aqueous media, efficient working, and competitive cost.^17,18^

Biological photocatalytic processes like natural photosynthesis evolved over billions of years, resulting in the Calvin cycle combined with effective photosensitizer/photon converter photosystems (PS1 and PS2). For efficient photocatalysis, the active molecule should harvest as many photons in the visible region as possible.^19^ This property is tunable in organic molecules via highest occupied molecular orbital (HOMO)/lowest unoccupied molecular orbital (LUMO) band gap engineering to fit the electrochemical window for a given process. Systems like TiO_2_ (3.2 eV band gap, UV-light irradiation) are much more challenging to modify to achieve a band gap alteration.^20^ Eosin Y is frequently used as a photosensitizer, although its fast photodegradation causes problems.^15,21^

**Figure 1:**
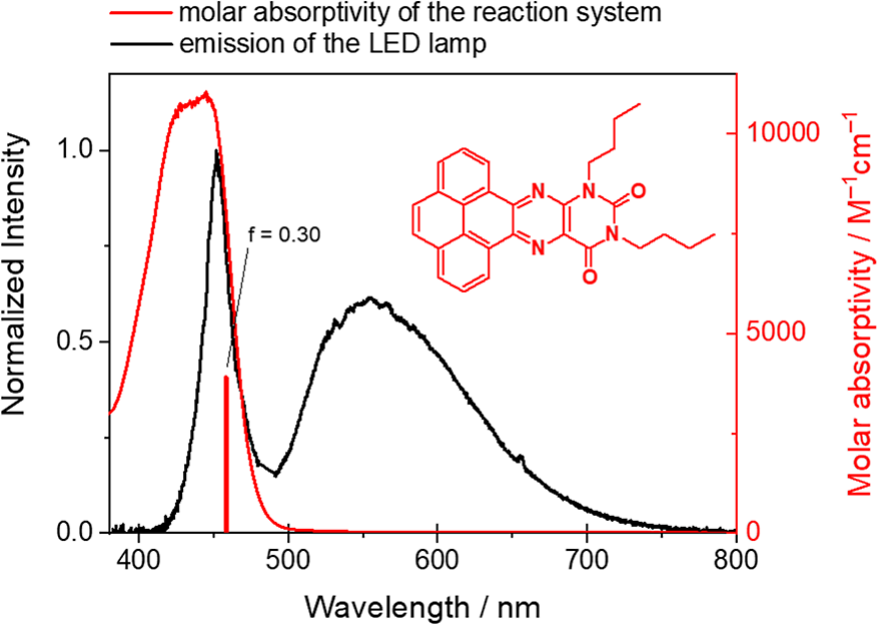
Molecular structure of the FP and the normalized spectrum of the light-emitting diode (LED) lamp used for irradiation, provided by the manufacturer—black, and the molar absorptivity of the reaction system containing FP (0.30 mM), K_2_PtCl_4_ (0.05 mM), and 8:1:1 (v/v/v) of the THF/water/triethylamine (TEA) solution (10.0 mL in total)—red. The vertical red line shows the computed HOMO/LUMO transition in THF. The letter f denotes the oscillator strength of the transition.

Alloxazines have attracted our attention as a part of the flavin family. Flavins are present in most biological systems, which take on diverse roles, from photosynthesis^22^ to soil detoxification.^23^ One outstanding example is vitamin B2, riboflavin,^24,25^ which contains the isoalloxazine tautomeric core. The change in visible light absorption has been achieved by fusing aromatic molecules to the pteridine moiety or substituting the nitrogen atoms (see Figure 1).^26,27^ While isoalloxazines are more frequent, the alloxazine isomer has superior photostability and a more straightforward synthesis. A previous study^28^ showed the variability of optical, electrochemical, and morphological properties of alloxazine derivatives. This property was applied by Golczak et al.^29^ in a fluorescence microscopy study of human red blood cells. Presently, flavins are used for the photocatalysis of various oxidative reactions—the preparation of sulfoxides^30^ or some cycloeliminations.^31^

Furthermore, previous electrochemical measurements performed by our group during studies of novel materials have shown favorable energy levels for hydrogen reduction. Because these molecules also have well-tunable visible-light absorption spectra, we have predicted using flavin-based systems for photocatalytic water splitting.^28,32^

Here in this paper, we report on utilizing novel fused flavin derivatives as photosensitizers for visible-light-driven hydrogen evolution reaction (HER). Our results open new possibilities in the realm of reductive photocatalysis, where the use of flavins remains rare 26.

## Results and Discussion

**Figure 2:**
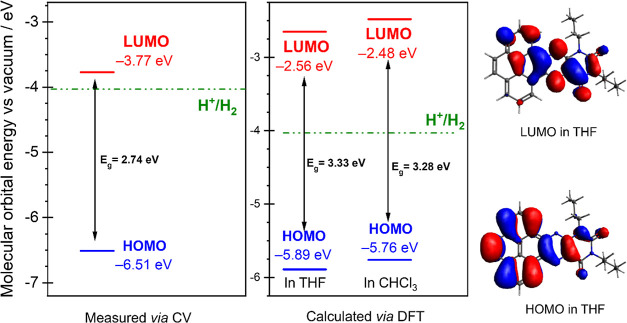
Energy of the FP’s molecular orbitals from CV measurements [conditions: 1 mM FP in CH_2_Cl_2_ with 0.1 M TBAPF_6_ and 0.1 mM ferrocene (internal standard). Scan rate 200 mV·s^–1^ 28] and TDDFT calculation in chloroform, and the computed MO energy levels in THF (corresponding to the optical HOMO/LUMO gap), along with visualizations of the orbitals. The green line shows the half-cell redox potential of H^+^/H_2_ (green) = −4.03 eV vs vacuum 19.

The molar absorption coefficient of the flavin photosensitizer (FP) is 10^5^ M^–1^ cm^–1^ at λ_abs,max_ = 450 nm, measured in the photocatalytic reaction solution (Figure 1) and suitable HOMO (−6.51 eV) and LUMO (−3.77 eV) levels indicating suitable band gap for use in artificial photosynthesis as a light harvester (Figure 2) 28. To better describe the FP molecule, theoretical calculations were done using density functional theory (DFT) to find the stable molecular geometry and frontier orbital energies. Afterward, time-dependent (TD) DFT was used to calculate the electronic transitions. The calculations were performed in implicit tetrahydrofuran (THF) solvent. A strong theoretical HOMO/LUMO transition was found at 458 nm, with an oscillator strength of 0.30. An inspection of Figure 1, where this transition is shown by a red vertical line, shows a good fit of the TDDFT theoretical transition to the experiment. Other significant transitions, with an oscillator strength above 0.01, were found outside of the area of interest, i.e., outside the emission spectrum of the lamp, and are summarized in Table S1.

Furthermore, Figure 2 shows the energy levels of the FP measured by cyclic voltammetry (CV) and calculated in implicit CHCl_3_ solvent in a previous study.28 Along with these, the presently calculated frontier molecular orbital (FMO) levels in THF are included. The FMO visualizations show the delocalization of the HOMO across the entire molecule, while the LUMO is located across the alloxazine moiety. In order to drive the reaction, the LUMO orbital of the photosensitizer must be above the redox potential of the H^+^/H_2_ couple 19 (denoted as a green line in Figure 1). However, the FP molecule must perform two processes in the context of this reaction, i.e., excitation and charge transfer.

**Figure 3:**
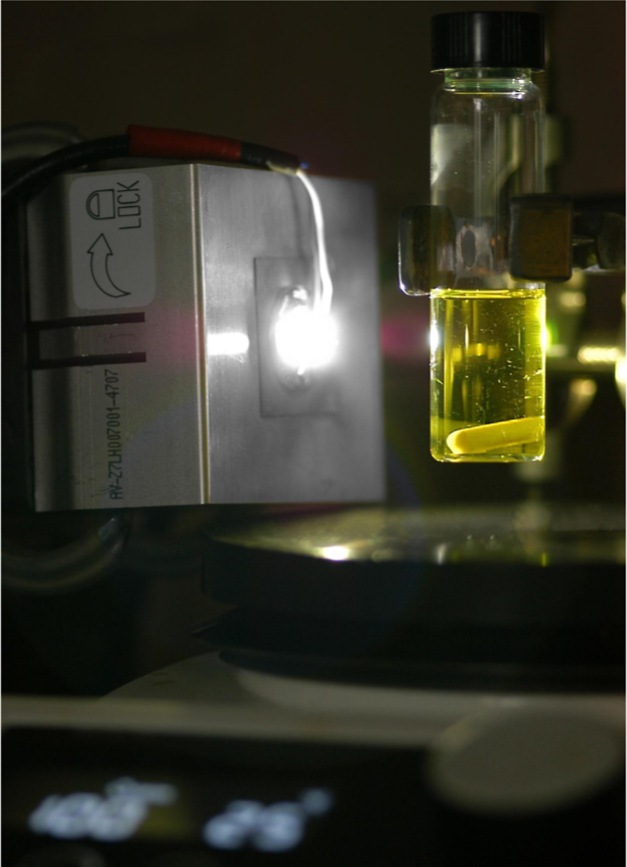
Picture of the reactor setup irradiated with an LED lamp.

For an insight into the fundaments and the mechanism of the process, we have decided on a partial water-splitting approach which required the use of a suitable electron-donating substance.^18,33^ Because of the very large work function,^34^ we have chosen platinum as the cocatalyst. Thus, the multicomponent catalytic system included a water-reduction cocatalyst (colloidal platinum) and TEA as a sacrificial electron donor (SD) to substitute the oxidation half-reaction. Even though the process is considered environmentally and economically unfavorable due to the requirement of SD consumption, it is useful to first examine photosensitizing properties straightforwardly before designing overall water-splitting systems.^19,35^ Light-driven H_2_ evolution took place in a glass vial reactor (Figure 3) and was triggered by visible light irradiation (λ > 400 nm) of the LED lamp. The emission spectrum of the lamp has a significant overlap with the FP’s absorption spectrum (Figure 1).

Pilot experiments showed that the system is capable of H_2_ evolution, and blank experiments indicated that H_2_ evolution could not occur without the FP or cocatalyst. The FP molecular structure cannot provide an active site for the H_2_ reduction itself; therefore, the presence of a cocatalyst was necessary. Similarly, no H_2_ production occurred in the dark (see Figure S1), and the process clearly requires light initiation. Therefore, we feel justified in claiming that we have prepared a novel photocatalytic system for hydrogen production.

**Figure 4:**
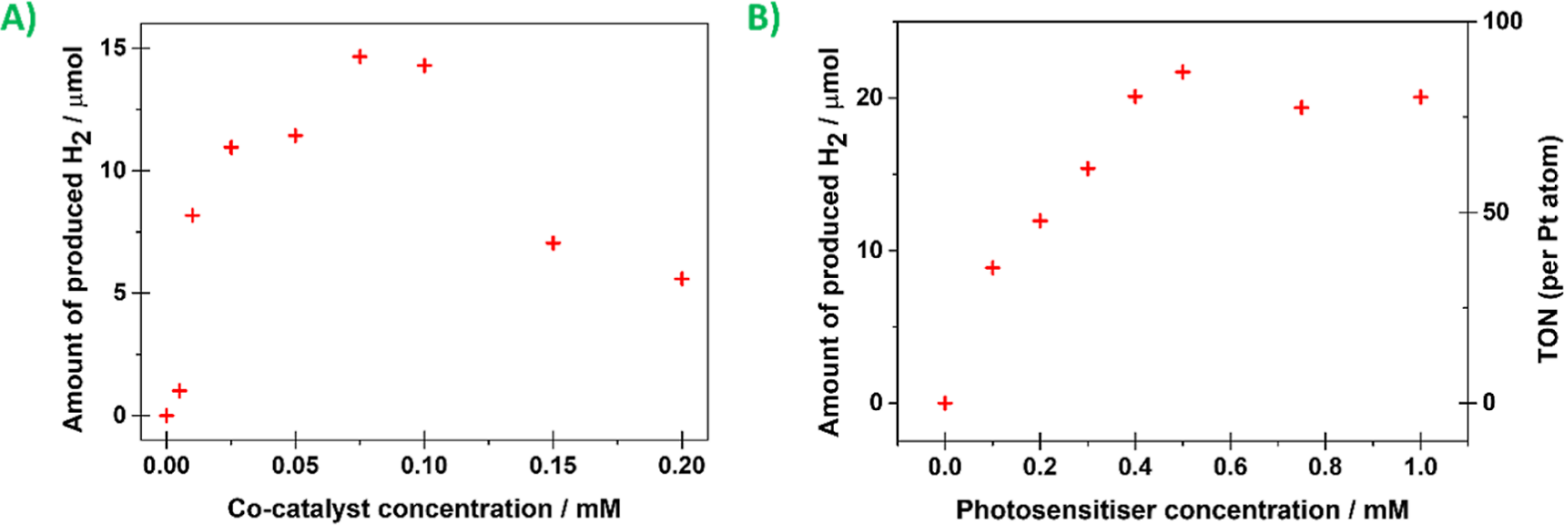
Photocatalytic measurements of 8:1:1 (v/v/v) of the THF/water/TEA solution (10.0 mL in total): (A) dependency of K_2_PtCl_4_ cocatalyst concentration in the reaction systems containing FP (0.30 mM), during 2 h of irradiation to the light-driven hydrogen evolution; (B) dependency of FP concentration in the reaction systems containing K_2_PtCl_4_ (0.05 mM), during 2 h of irradiation to the light-driven hydrogen evolution.

At first, experiments with different concentrations of cocatalyst K_2_PtCl_4_ and FP were performed. By these experiments, the optimal cocatalyst concentration was revealed to be 0.05 mM. The optimal cocatalyst concentration was revealed by varying the K_2_PtCl_4_ concentrations and vice versa. The measurements with different K_2_PtCl_4_ concentrations are depicted in Figure 4-A. The maximum H_2_ evolution of 14.7 μmol was achieved for 0.30 mM FP and 75 μM K_2_PtCl_4_ after 2 h of irradiation. We have noticed a downtrend at high platinum concentrations, which can be assigned to formation of the in situ-generated colloidal platinum aggregates, as is often discussed in the literature.^36,37^ The H_2_ evolution dependence on FP concentration is shown in Figure 4-B. The turnover number (TON) linearly increased with FP concentration and then reached 87 at 0.50 mM FP and saturated.

Further rise of the FP’s amount had no significant effect on the H_2_ evolution. Probably, due to the fact that the light from the LED is absorbed, the excess FP has no way to affect the photolysis. However, at low FP concentrations, the H_2_ evolution is directly proportional to FP concentration, suggesting that the photolysis is FP-limited. To further illustrate this trend, Figure S2 shows the fit of Figure 4B with the equation H_2_ = *a*(1 – 10^–*bx*^), where *a* corresponds to a proportionality constant, *b* corresponds to the product of molar absorptivity (M^–1^ cm^–1^) and optical length of the reactor (cm), and *x* corresponds to the FP concentration (mM).

Regarding performance and cost efficiency, the optimal ratio of the FP to cocatalyst is 1:6 by 0.30 mM photosensitizer concentration. The TON was calculated based on the number of single-electron transfer processes using an equation
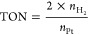
where *n*_H_2__ represents the amount of evolved hydrogen (μmol), and *n*_Pt_ represents the amount of the cocatalyst (μmol).

**Figure 5:**
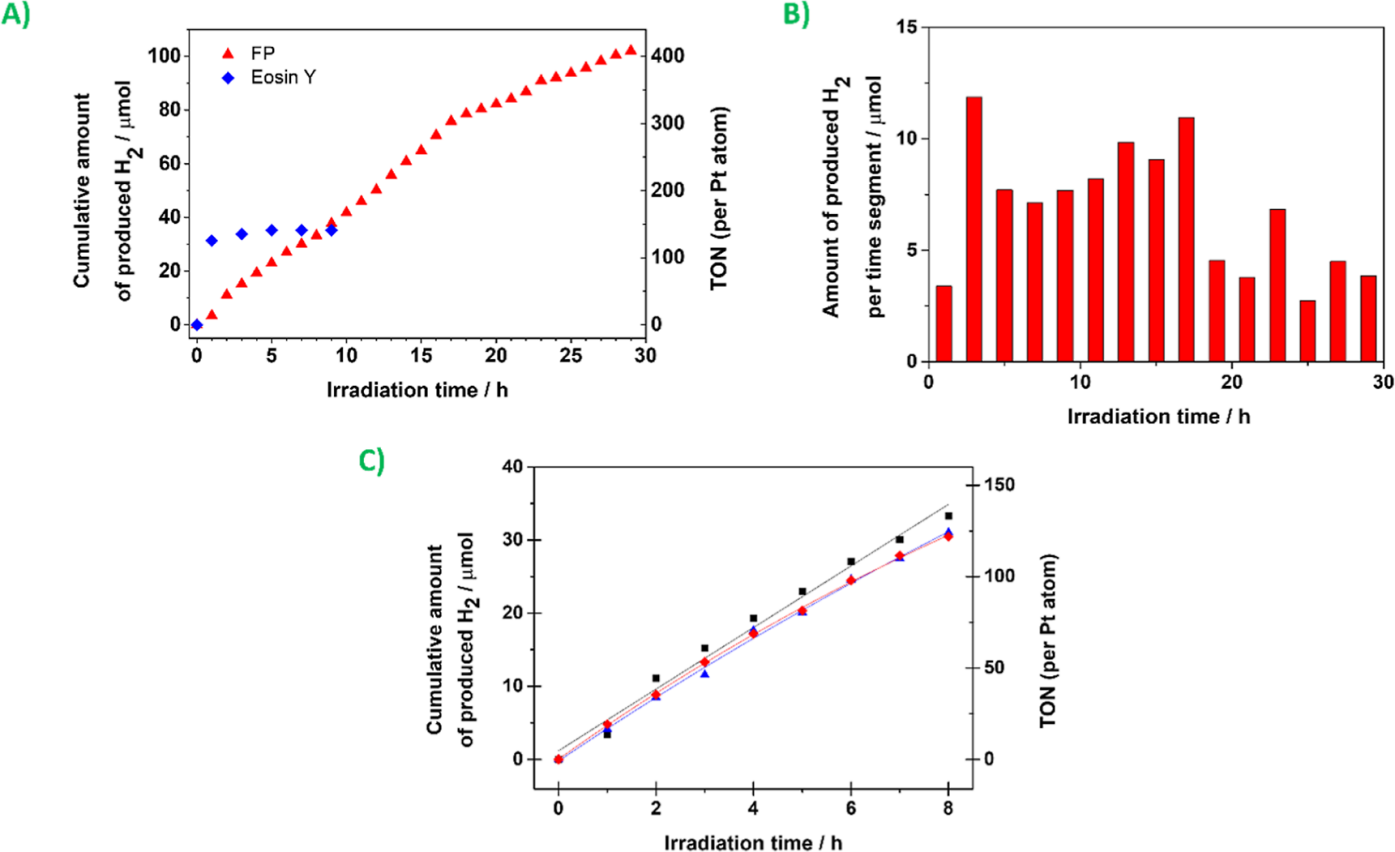
Photocatalytic measurements of the reaction system containing FP (0.30 mM), K_2_PtCl_4_ (0.05 mM), and 8:1:1 (v/v/v) of the THF/water/TEA solution (10.0 mL in total): (A) time dependency of light-driven H_2_ evolution and (B) amount of H_2_ evolved per time segment during the same experiment; (C) three separated measurements of the time dependency during 8 h of irradiation.

Afterward, the photostability of the FP and reproducibility of the process within our system were studied. As a result, we have confirmed the steady H_2_ evolution for 29 h (Figure 5A). The aggregation of the cocatalyst possibly caused the visible decrease at the 18th hour, as we noticed a dark precipitate was formed (Figure 5B), which we considered to be the catalytically inactive platinum black.^38,39^ After the experiment, the UV–vis measurement of the FP showed no significant changes to the pristine state. Therefore, we believe no photodegradation of the material occurred (see Figure S3). Moreover, the photocatalytic properties of the FP were compared with Eosin Y, which showed rapid photodegradation, as previously documented,^15,21^ followed by a consequent dramatic decrease in H_2_ evolution.

Last but not least, three simultaneous 8 h measurements (Figure 5C) were carried out that showed suitable reproducibility of the measurements.

### Conclusions and Outlooks

Our experiments demonstrate the use of a novel flavin derivative acting as a photosensitizer compared to Eosin Y for water splitting and HER. The system works with the highest efficiency when the concentration of the FP is 0.5 mM. The optimal cocatalyst K_2_PtCl_4_ ratio was determined to be 1:6. This system was capable of continuous hydrogen production without a significant decline over 18 h, indicating sufficient photostability and reproducibility by repeated measurements. Moreover, we have proved that the system can work even under low irradiation in comparison to, e.g., the use of the solar simulator or xenon lamp.

These pilot experiments can serve as a springboard for future, more comprehensive work, which includes several other applications, such as the reduction of CO_2_ to methane or alcohols. Furthermore, to increase the attractiveness and competitiveness of photocatalytic processes, the reliance on sacrificial reagents will have to be eliminated to simplify the overall material balance and stop the use of potentially harmful chemicals.

### Experimental Section

Commercially available chemicals were used as received. ^1^H- and ^13^C NMR spectra were recorded in CDCl_3_ using a Bruker AVANCE III 500 MHz spectrometer (Bruker, BioSpin GmbH, Germany) with working frequencies of 500 and 126 MHz, respectively, at 30 °C. Chemical shifts are expressed in parts per million (δ scale) downfield from tetramethylsilane and are referenced to residual protium in the NMR solvent (CHCl_3_: δ7.25 ppm). Coupling constants (*J*) are given in Hz with coupling expressed as d—doublet, dd—doublet of doublet, t—triplet, ddd—doublet of doublet of doublets, p—pentet, and m—multiplet. Melting points were determined using the Kofler apparatus with a microscope Nagema PHMK 05 (Nagema, Dresden, Germany).

### 10,12-Dibutylpyreno[4,5-g]pteridine-11,13(10*H*,12*H*)-dione (FP)


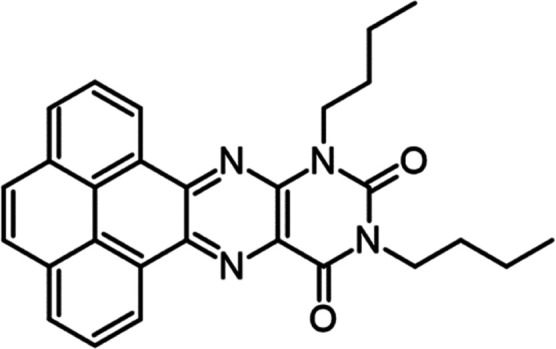


### Molecular Mass: 450.53 g·mol^–1^

^1^H NMR (CDCl_3_, 500 MHz): δ 9.30 (dd, *J* = 7.9, 1.4 Hz, 1H), 9.07 (dd, *J* = 8.2, 1.4 Hz, 1H), 8.62 (d, *J* = 8.2 Hz, 1H), 8.58 (dd, *J* = 8.2, 1.4 Hz, 1H), 7.86 (ddd, *J* = 8.2, 7.0, 1.4 Hz, 1H), 7.81–7.72 (m, 3H), 4.59–4.52 (m, 2H), 4.27–4.19 (m, 2H), 1.89 (p, 2H), 1.79 (p, 2H), 1.61–1.44 (m, 4H), 1.06 (t, *J* = 7.4 Hz, 3H), 1.01 (t, *J* = 7.4 Hz, 3H).

^13^C NMR (CDCl_3_, 126 MHz): δ 159.7, 150.4, 145.7, 144.0, 138.7, 131.3, 131.2, 130.0, 128.4, 128.2, 127.8, 127.7, 127.2, 127.0, 126.9, 126.7, 126.6, 124.8, 124.2, 123.7, 42.7, 42.5, 30.1, 29.9, 20.5, 20.4, 14.1, 14.0.

### Melting Point: 240–241 °C

The irradiation was mediated by an MCPCB-Mounted MCWHD3 LED (Thorlabs, Newton, New Jersey, USA) with a typical output power of 2700 mW. The irradiance inside the glass vial was measured by using a Gigahertz-Optik X97 radiometer (probe calibrated for an interval of 400–800 nm). The irradiance value was 8.32 mW cm^–2^. Gaseous samples were taken using a gastight syringe equipped with a sampling syringe valve system (Hamilton Company, Reno, Nevada, USA). Gas chromatography analysis for H_2_ was performed on a TRACE 1300 (Thermo Scientific, Waltham, Massachusetts, USA) with a 5 Å molecular sieve column (Thermo Scientific, Waltham, Massachusetts, USA), thermal conductivity detector (Thermo Scientific, Waltham, Massachusetts, USA), and nitrogen carrier gas. H_2_ production was quantified from a one-point calibration of the H_2_/N_2_ mixture.

The absorption spectra were measured using a SPECORD 50 PLUS UV–vis spectrophotometer (Analytik Jena, Jena, Germany). First, the absorption spectra were corrected to a baseline, which was determined by the measurement of absorption spectra of the THF/deionized water (8:1 ratio) solvent.

## Materials and Synthesis

The molecule in question is the 10,12-dibutylpyreno[4,5-g]pteridine-11,13(10*H*,12*H*)-dione further referred to as FP was prepared according to the procedure in ref (28), characterized with NMR and melting point measurements (summarized below) and used without further purification. All other solvents and reagents were obtained commercially and used without further purification.

### 2 h Net Hydrogen Evolution Experiments

A 25 mL photoreactor equipped with a magnetic stirrer and enclosed using a cap with a septum was filled with 10 mL of THF/deionized water (8:1) reaction solution containing various concentrations of the FP, various concentrations of K_2_PtCl_4_, and TEA (1 mL). The solution was degassed with nitrogen and irradiated for 2 h. Afterward, a sample of the atmosphere was taken manually with a gastight syringe. All measurements were performed under oxygen-free conditions and at ambient temperature.

### TD H_2_ Evolution Measurements

A 25 mL photoreactor equipped with a magnetic stirrer and enclosed using a cap with a septum was filled with 10 mL of THF/deionized water (8:1) reaction solution containing various concentrations of the FP, various concentrations of K_2_PtCl_4_, and TEA (1 mL). The solution was degassed with nitrogen and was irradiated for an hour. Afterward, a sample of the atmosphere was taken manually with a gastight syringe. The degassing, irradiation, and sampling procedure was repeated for the same solution after every hour of the measurement. All measurements were performed under oxygen-free conditions and at ambient temperature.

### Blank Experiments with Irradiation

A 25 mL photoreactor equipped with a magnetic stirrer and enclosed using a cap with a septum was filled with 10 mL of THF/deionized water (8:1) reaction solution containing either 0.3 mM FP and TEA (1 mL) or 0.05 mM K_2_PtCl_4_ and TEA (1 mL). The solution was degassed with nitrogen and irradiated for 2 h. Afterward, a sample of the atmosphere was taken manually with a gastight syringe. All measurements were performed under oxygen-free conditions and at ambient temperature.

### Blank Experiments without Irradiation

A 25 mL photoreactor equipped with a magnetic stirrer and enclosed using a cap with a septum was filled with 10 mL of THF/deionized water (8:1) reaction solution containing various concentrations of the FP, various concentrations of K_2_PtCl_4_, and TEA (1 mL). The solution was degassed with nitrogen and was left in the dark for 2, 5, 10, and 15 h. Afterward, a sample of the atmosphere was taken manually with a gastight syringe. All measurements were performed under oxygen-free conditions and at ambient temperature. The results of this experiment can be found in the Supporting Information (Figure S1).

## Computational Details

The quantum chemical calculations were performed using the Gaussian 16 program package^40^ (see Table S1). The optimal geometries of the FP and Eosin Y were found via DFT calculation on the B3LYP^41^ level of theory without any constraints (an energy cutoff of 10^–5^ kJ mol^–1^, final RMS energy gradient under 0.01 kJ mol^–1^ A^–1^). Furthermore, the 6-31+G** basis set was used^42^ and proven effective in the past.^43^ The density-based solvation model^44^ was used to account for the solvent influence, while dispersion interactions were treated with Grimme’s corrections (GD3BJ).^45^ Since THF is the primary solvent in the mixture, it was used as the only solvent in the computations. Furthermore, the vertical singlet transition energies and oscillator strengths were found by using the TDDFT method with the lowest-energy conformations of the given molecules used as initial states. The potential energy surface minima were confirmed by inspecting the frequencies (no imaginary frequencies were found).

## Supporting Information Available

Significant theoretical ground-state to excited-state transitions of the FP molecule, photocatalytic measurements of the reaction system in the dark, exponential fit of the amount of evolved hydrogen where *a* corresponds to a proportionality constant, and UV–vis of the FP before and after (recovered from the reaction system) (PDF)
