# Supplementary material for: Nature-Inspired Photocatalytic Hydrogen Production with a Flavin Photosensitizer
Source: ACS Omega. 2024 Jan 26;9(5):5534–40. doi: 10.1021/acsomega.3c07458 (PMC10851229; doi:10.1021/acsomega.3c07458)
Supplement: Supplementary file 1 — ao3c07458_si_001.pdf [file ao3c07458_si_001.pdf]

Nature-Inspired Photocatalytic Hydrogen Production with Flavin Photosensitizer

Supporting Information

Lucia Ivanová[1], Jan Truksa[1], Dong Ryeol Whang[2], Niyazi Serdar Sariciftci[3], Cigdem Yumusak[3], Jozef Krajčovič[1*]*

1. Faculty of Chemistry, Brno University of Technology, Purkyňova 118, CZ-612 00 Brno, Czech Republic

2. Department of Advanced Materials, Hannam University, 70 Hannamro, Daedeok-Gu Daejeon 34430, Republic of Korea

3. Linz Institute for Organic Solar Cells (LIOS), Institute of Physical Chemistry, Johannes Kepler University Linz, Altenberger Straße 69, 4040 Linz, Austria

*Corresponding authors:

krajcovic@fch.vut.cz (J. Krajčovič)

phone: +420541149433

cigdem.yumusak@jku.at (C. Yumusak)

phone: +43 732 2468 5847

Table S1: The significant (oscillator strength above 0.01) theoretical ground-state to excited-state transitions of the FP molecule.

Transition Number	Energy / eV	Wavelength / nm	Oscillator strength	Transition
1	2.705	458. 4	0.30	HOMO → LUMO
2	3.299	375.8	0.01	HOMO – 1 → LUMO
3	3.520	353.2	0.16	HOMO – 3 → LUMO
4	3.522	352.0	0.50	HOMO → LUMO + 1
5	3.664	338.4	0.21	HOMO – 2 → LUMO
6	3.835	323.3	0.06	HOMO – 1 → LUMO + 1
8	4.116	301.2	0.06	HOMO → LUMO + 3
9	4.231	293.0	0.29	HOMO → LUMO + 3
10	4.328	286.5	0.82	HOMO – 4 → LUMO
11	4.426	280.1	0.03	HOMO – 5 → LUMO
12	4.446	278.9	0.03	HOMO – 2 → LUMO + 1

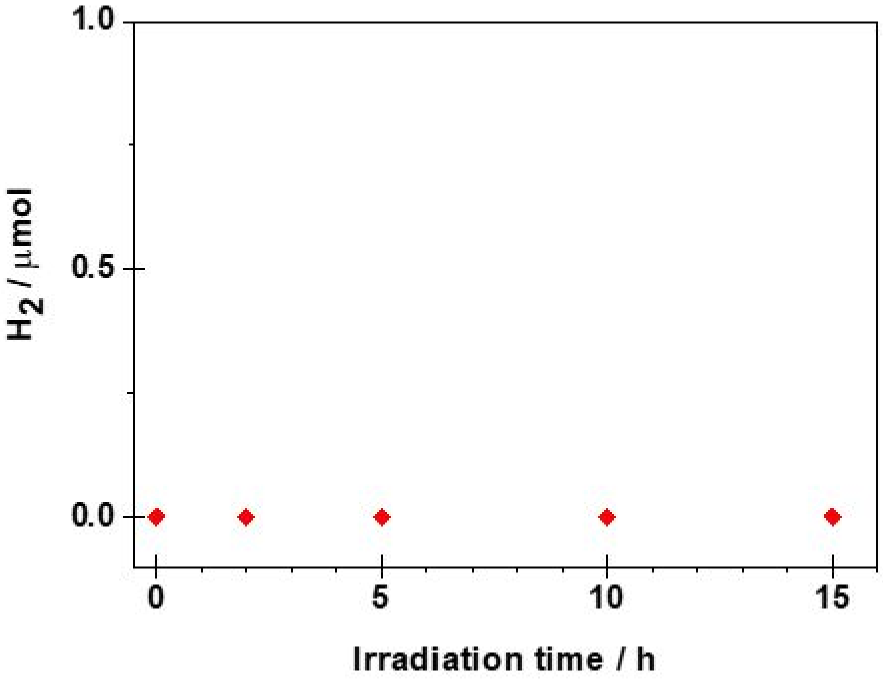

Figure S1: Photocatalytic measurements of the reaction system containing FP (0.30 mM), K_2PtCl_4 (0.05 mM) and 8:1:1 (v/v/v) of the THF/water/TEA solution (10.0 ml in total) in the dark.

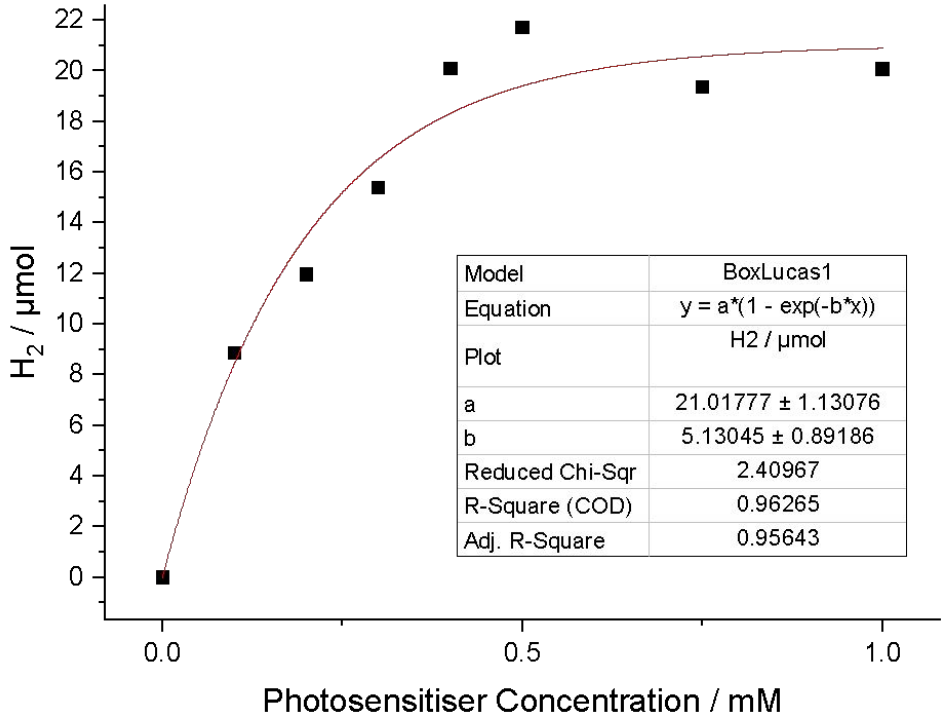

Figure S2: The exponential fit of the amount of evolved hydrogen where a corresponds to a proportionality constant, b corresponds to the product of molar absorptivity (M^{-1} cm^{-1}) and optical length of the reactor (cm), and x corresponds to the PS concentration (mM).

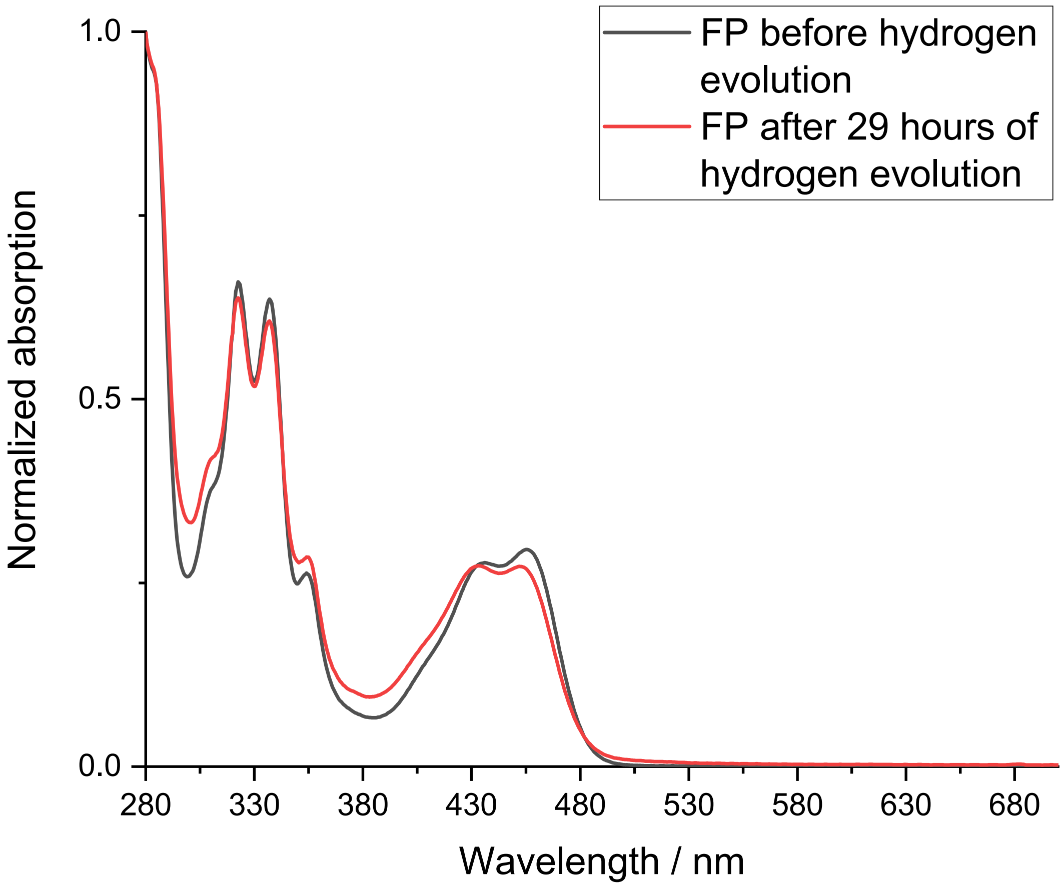

Figure S3: UV-VIS of PS before and after (recovered from the reaction system).
